# Supplementary material for: A Novel Bionebulizer Approach to Study the Effects of Natural Mineral Water on a 3D In Vitro Nasal Model from Allergic Rhinitis Patients
Source: Biomedicines. 2024 Feb 9;12(2):408. doi: 10.3390/biomedicines12020408 (PMC10886703; doi:10.3390/biomedicines12020408)
Supplement: Supplementary file 1 [file biomedicines-12-00408-s001.zip › biomedicines-2848583-supplementary-new.pdf]

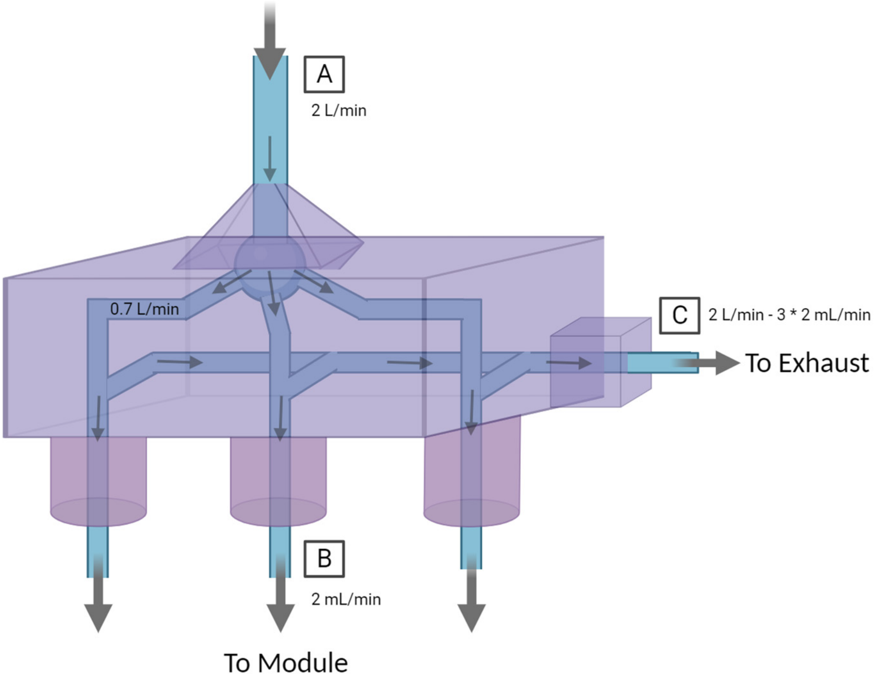

Figure S1. The flux rate generated by the aerosol generator was 2 L/min (**A**) and it is split into three channels (2.0 L/min/3, i.e., 0.7 L/min). The flux rate that goes to the cell inserts in the exposure model system is determined by the vacuum, and is 2 mL/min (**B**). Most of the remaining aerosol is exhausted, i.e., 2 L/min minus 3 times 2 mL/min (**C**). Created with BioRender.com.

Table S1. Estimation of flow rate in *in vivo* 0.33 cm^2 human nasal epithelium.

Flow rate range	6 L/min[a]		12 L/min[b]		60 L/min in non-invasive therapy[c]		70 L/min non-invasive therapy[d]	
Total nasal area[e,f]	150 cm^2	160 cm^2	150 cm^2	160 cm^2	150 cm^2	160 cm^2	150 cm^2	160 cm^2
Estimation flow rate per 0.33 cm^2*	13mL/min	12mL/min	26mL/min	25mL/min	132mL/min	124mL/min	154L/min	144mL/min
Flow rate ratio**	6.5 x	6.0 x	13.0 x	12.5 x	66.0 x	62.0 x	77.0x	80.0x

[a][40]; [b][41]; [c][42]; [d]https://www.fphcare.com/ [e][43]; [f][15]. *Estimation average flow rate per 0.33 cm^2, considering that 150 cm^2 or 160 cm^2 contains 455 or 485 areas of 0.33 cm^2 (150/0.33=455 or 160/0.33=485), respectively. **Estimation flow rate per 0.33 cm^2/This study (2.0mL/min/0.33cm^2).

References

a) 40. Hall, J.E. Guyton and Hall Textbook of Medical Physiology, 13th ed.; Elsevier: Amsterdam, The Netherlands, 2016; ISBN 2013206534.
b) 41. Kumar, H.; Jain, R.; Douglas, R.G.; Tawhai, M.H. Airflow in the Human Nasal Passage and Sinuses of Chronic Rhinosinusitis Subjects. PLoS ONE 2016, 11, e0156379. https://doi.org/10.1371/journal.pone.0156379.
c) 42. Burrowes, K.S.; De Backer, J.; Kumar, H. Image-based Computational Fluid Dynamics in the Lung: Virtual Reality or New Clinical Practice? WIREs Syst. Biol. Med. 2017, 9, e1392. https://doi.org/10.1002/wsbm.1392.
e) 43. Mygind, N.; Dahl, R. Anatomy, Physiology and Function of the Nasal Cavities in Health and Disease. Adv. Drug Deliv. Rev. 1998, 29, 3–12. https://doi.org/10.1016/S0169-409X(97)00058-6.
f) 15. Gizurarson, S. Anatomical and Histological Factors Affecting Intranasal Drug and Vaccine Delivery. *Curr. Drug Deliv.* **2012**, *9*, 566–582. https://doi.org/10.2174/156720112803529828.
